# Assimilation of Cholesterol by *Monascus purpureus*

**DOI:** 10.3390/jof6040352

**Published:** 2020-12-09

**Authors:** Theresa P. T. Nguyen, Margaret A. Garrahan, Sabrina A. Nance, Catherine E. Seeger, Christian Wong

**Affiliations:** Department of Chemistry & Biochemistry, Loyola University Maryland, Baltimore, MD 21210, USA; magarrahan@loyola.edu (M.A.G.); sanance@loyola.edu (S.A.N.); ceseeger@loyola.edu (C.E.S.); ckwong@loyola.edu (C.W.)

**Keywords:** *M. purpureus*, red yeast rice, filamentous fungi, cholesterol reduction, probiotic potential

## Abstract

*Monascus purpureus*, a filamentous fungus known for its fermentation of red yeast rice, produces the metabolite monacolin K used in statin drugs to inhibit cholesterol biosynthesis. In this study, we show that active cultures of *M. purpureus* CBS 109.07, independent of secondary metabolites, use the mechanism of cholesterol assimilation to lower cholesterol in vitro. We describe collection, extraction, and gas chromatography-flame ionized detection (GC-FID) methods to quantify the levels of cholesterol remaining after incubation of *M. purpureus* CBS 109.07 with exogenous cholesterol. Our findings demonstrate that active growing *M. purpureus* CBS 109.07 can assimilate cholesterol, removing 36.38% of cholesterol after 48 h of incubation at 37 °C. The removal of cholesterol by resting or dead *M. purpureus* CBS 109.07 was not significant, with cholesterol reduction ranging from 2.75–9.27% throughout a 72 h incubation. Cholesterol was also not shown to be catabolized as a carbon source. Resting cultures transferred from buffer to growth media were able to reactivate, and increases in cholesterol assimilation and growth were observed. In growing and resting phases at 24 and 72 h, the production of the mycotoxin citrinin was quantified via high-performance liquid chromatography-ultraviolet (HPLC-UV) and found to be below the limit of detection. The results indicate that *M. purpureus* CBS 109.07 can reduce cholesterol content in vitro and may have a potential application in probiotics.

## 1. Introduction

*Monascus purpureus* is a filamentous fungus that produces a variety of secondary metabolites, including pigments, lipids, and monacolins. *M. purpureus* is most widely known for the fermentation of white rice to produce a deep red rice known as angkak or beni koji [1,2,3]. More commonly, the fermented product is called “red yeast rice”, though *Monascus* species are more accurately molds. *M. purpureus* fermented rice is used in food preparation for flavoring, coloring, and preservation, and is also consumed in traditional Chinese medicine to improve ailments of circulation and heart health [2,4,5,6,7]. Modern research explored the health claims, and found a plausible cause: *M. purpureus* can synthesize monacolins, naturally occurring compounds capable of decreasing cholesterol levels by inhibiting HMG-CoA reductase, the rate-limiting step in cholesterol biosynthesis [7,8]. The most potent of the *Monascus* monacolins, monacolin K, was isolated and patented as lovastatin and is widely prescribed to treat hypercholesterolemia [8]. Statins are effective treatments for high cholesterol; however, side effects, low tolerance, and the cost of the drug have led patients to pursue alternative options to lower their cholesterol levels [9]. As lyophilized red yeast rice (RYR) supplements emerged as a naturopathic alternative in the U.S., the Food and Drug Administration (FDA) restricted the market, determining that without standardization and quality control for the amount of monacolin K, RYR was not a dietary supplement, but rather an unauthorized new drug [5,10]. As a result, current U.S. supplements marketed as RYR may contain, at most, only trace amounts of monacolin K [5,10,11,12]. Nevertheless, several clinical studies report that RYR supplements may be an effective treatment option for hypercholesterolemia as significant reductions in low-density lipoprotein (LDL) cholesterol and total cholesterol levels were observed in patients taking RYR [4,10,11,12,13,14].

Two-thirds of cholesterol required for cell membranes and biosynthesis of steroid hormones and bile acids is endogenously synthesized; however, an excess of cholesterol is a major risk factor for cardiovascular disease [15,16,17]. The World Health Organization (WHO) projects that by 2030, nearly 23.6 million people will die from cardiovascular disease, which includes heart disease and stroke as the leading causes of death [18]. Research into the efficacy of therapies such as RYR supplements in the treatment of elevated cholesterol levels is ongoing and critical for treating cardiovascular disease.

*M. purpureus* strains administered via RYR supplements in clinical trials often have unspecified viability [19,20]. This led us to ask if living *M. purpureus*, and not simply its secondary metabolites, have cholesterol-lowering properties. Several studies have demonstrated that active probiotic microorganisms introduced into intestinal microbiota can improve the overall lipid content in human blood serum [15,21,22,23,24,25]. Probiotics are defined as live microorganisms that, when administered in adequate amounts, can confer a health benefit on the host [26]. The fermented product of *M. purpureus* can be labeled as “contains live and active cultures,” but clinical studies on the safety, viability, and dosage of live *M. purpureus* strains are still necessary before characterizing strains of *Monascus* as probiotic [26,27,28]. Additionally, factors like mycotoxins should considered; *Monascus* species, like *Aspergillus* and *Penicillium*, naturally synthesize the cytotoxic citrinin at low levels [29,30]. In the European Union (EU) and US, *Monascus* pigments are prohibited from use in food industries [3,31,32]. Still, many *Monascus* strains are generally regarded as safe (GRAS) in many Asian countries and the commercial interest in fermentation has led to easy access of RYR outside of Asia [6,11,33].

While the cholesterol-lowering benefits of probiotics have been highlighted in in vivo studies, the mechanisms of probiotics remain not fully understood [34,35,36]. Potential mechanisms have been proposed in vitro, such as the removal of cholesterol from media by the assimilation or uptake of cholesterol by probiotic strains of *Lactobacilli* and *Bifidobacteria* [35,37,38,39,40,41]. The ability of cholesterol removal appears to be growth- and strain-specific, with nearly all research focused on bacterial strains [35,36,37,38,39,40,41]. We anticipate that as beneficial fungi are characterized, greater attention will be drawn to the roles of fungi in health, nutrition, and the mycobiome. Currently, *Saccharomyces boulardii* is the only fungus with a strain commercially labeled in the U.S. as a probiotic, as it can survive the passage through the gastrointestinal tract, present good growth at 37 °C, complement treatment of gastrointestinal diseases, and also assimilate cholesterol [37,42,43,44,45,46].

In this study, we evaluated the ability of the fungus *M. purpureus* CBS 109.07 to assimilate cholesterol in vitro. We developed new sample collection methods and used gas chromatography to quantify the levels of cholesterol remaining after incubation with *M. purpureus* CBS 109.07. Our data indicate that this strain of *M. purpureus* is capable of cholesterol assimilation, a cholesterol-lowering mechanism separate from its ability to produce monacolins and which has not been previously reported. The present study does not establish the safety and efficacy of CBS 109.07 as a therapeutic agent, however, these results lay the groundwork for the possibility that active *M. purpureus* CBS 109.07 may have probiotic potential based on its ability to assimilate cholesterol.

## 2. Materials and Methods

### 2.1. Strains and Media Conditions

*M. purpureus* Went teleomorphic type strain CBS 109.07 (ATCC 16365) was obtained from the American Type Culture Collection (ATCC) strain bank and chosen for its ability to grow at 30 °C. *M. purpureus* CBS 109.07 has been used in food-grade studies for human and animal food applications [6,47,48]. *M. purpureus* was grown in a malt extract media (MEA) containing: 2% soluble Bactomalt extract (BD Bioscience), 2% glucose, 1% peptone, and pH adjusted to pH 7. Plate media contained 2% agar. Phosphate-buffered saline (PBS) solution contained 8.0 g NaCl, 0.2 g KCl, 1.44 g Na_2_HPO_4_, 0.24 g KH_2_PO_4_ for every 1.0 L solution and was pH adjusted to pH 7. Where indicated, PBS was supplemented with 6.72 g/L of yeast nitrogen base without amino acids (BD Difco) or 5 g/L ammonium sulfate, and pH was adjusted to 7 before sterilization. Bile salt supplemented media contained 0.3% (*w*/*v*) oxgall (BD Difco).

### 2.2. Submerged Culture Preparation

A sterilized 5 mm cork-borer was used to remove an agar plug of *M. purpureus* CBS 109.07 grown on MEA plate media. The agar plug was subcultured into 4 mL of liquid MEA media and incubated at 30 °C. After four day incubation at 30 °C at 150 rpm, a colorless, spherical fungal pellet was formed. The pellet was transferred into new media with 0.3% oxgall at 37 °C and 60 rpm for growth curve, cholesterol assimilation, and citrinin production experiments.

### 2.3. Cholesterol Assimilation

#### 2.3.1. Cholesterol Reagents

A stock solution of cholesterol (Lipids Cholesterol Rich from adult bovine serum; Sigma-Aldrich, St. Louis, MO, USA) at 10 mg/mL was used to prepare cholesterol assimilation assays and to prepare a 6-point calibration curve as described in Section 2.3.6 [37]. A stock solution of 5-α-cholestane (Sigma-Aldrich, St. Louis, MO, USA) at 2.5 mg/mL was used as an internal standard in the lipid extractions.

#### 2.3.2. Culture Preparation for Growing, Resting, Dead, and Control Conditions

Growing, resting, and *M. purpureus* control conditions contained *M. purpureus* CBS 109.07 pellets that were homogenized using a sterilized glass douncer in a sterile 50 mL conical tube and divided into replicates. Dead culture conditions contained *M. purpureus* CBS 109.07 pellets that were autoclaved at 121 °C for 20 min under 15 psi pressure and transferred to fresh media. Growing and dead cultures contained 10 mL MEA media in sterile 50 mL borosilicate tubes; resting cultures contained 10 mL of PBS in sterile 50 mL borosilicate tubes; resting cultures supplemented with nitrogen sources contained 10 mL of PBS with ammonium sulfate or 10 mL of PBS with yeast nitrogen base. Cholesterol assimilation and dry weight experiments were supplemented with 0.3% oxgall and incubated at 37 °C and 60 rpm. With the exception of the *M. purpureus* control, all conditions were incubated with 120 µg/mL cholesterol. Media control without *M. purpureus* contained 120 µg/mL cholesterol and 0.3% (*w*/*v*) oxgall in 10 mL MEA.

#### 2.3.3. Cholesterol Assimilation and Dry Weight Growth Curve

Cholesterol assimilation and dry weight experiments for each growth condition were prepared in triplicate, where three independent sets of culture were homogenized and divided into six sterile 50 mL borosilicate tubes to account for six timepoints (0, 24, 36, 48, 60, and 72 h). At designated time point, a 1.0 mL aliquot of culture supernatant was collected, centrifuged at 2000× *g* for 15 min, and stored in a 50 mL borosilicate glass tube with a PTFE-lined cap at −20 °C. The remaining 9.0 mL of the culture was then harvested and filtered via vacuum flask and a pre-weighed Whatman filter #1. The contents were allowed to air dry for five days and dry weight was measured on an analytical balance.

#### 2.3.4. *M. Purpureus* Dormancy Experiment

Cholesterol assimilation and dry weight experiments were prepared in triplicate, where three independent sets of cultures were homogenized and divided into five sterile 50 mL borosilicate tubes to account for five timepoints (0, 24, 48, 72, and 96 h). *M. purpureus* was incubated in PBS buffer, pH 7 with 0.3% oxgall and 120 µg/mL cholesterol. At designated time points, a 1.0 mL aliquot of culture supernatant was collected, centrifuged at 2000× *g* for 15 min, and stored in a 50 mL borosilicate glass tube with a PTFE-lined cap at −20 °C. *M. purpureus* samples were then washed twice under sterile conditions with 10 mL of PBS + 0.3% oxgall, and transferred to 10 mL MEA media with 0.3% oxgall and 120 µg/mL cholesterol. A 1.0 mL aliquot of culture supernatant was collected at the starting point (t = 0 h), and also at day 4 and day 7 of incubation in MEA media. At day 7, the culture was harvested and filtered via vacuum flask and a pre-weighed Whatman filter #1. The contents were allowed to air dry for five days and dry weight was measured on an analytical balance.

#### 2.3.5. Resting *M. Purpureus* Supplemented with Nitrogen Sources Experiment

Cholesterol assimilation and dry weight experiments were prepared in duplicate, where two independent sets of cultures were homogenized and divided into three sterile 50 mL borosilicate tubes to account for three timepoints (0, 24, and 72 h). *M. purpureus* was incubated in PBS buffer supplemented with either ammonium sulfate or yeast nitrogen base without amino acids, pH 7 with 0.3% oxgall and 120 µg/mL cholesterol. At designated time points, a 1.0 mL aliquot of culture supernatant was collected, centrifuged at 2000× *g* for 15 min, and stored in a 50 mL borosilicate glass tube with PTFE lined cap at −20 °C. *M. purpureus* samples were then washed twice under sterile conditions with 10 mL of PBS + 0.3% oxgall, and transferred to 10 mL MEA media with 0.3% oxgall. At day 4, the culture was harvested and filtered via vacuum flask and a pre-weighed Whatman filter #1. The contents were allowed to air dry for five days and dry weight was measured on an analytical balance.

#### 2.3.6. Cholesterol Extraction 

Cholesterol assimilation samples were thawed and a stock solution of cholesterol (10 mg/mL) was used to prepare cholesterol standards in a range from 10–150 µg/mL in a borosilicate glass tube. To each sample and standard, 20 µL of 2.5 mg/mL internal standard 5-α-cholestane was added. Direct saponification was carried out on all samples and standards based on the method described by Fletouris et al. [49]. Four millimeters of methanolic 0.5 M KOH solution was added to each tube which was then capped and vortexed for 15 s. The samples and standards were heated for a total of 15 min in an 80 °C water bath, and removed every 5 min to vortex for 10 s. After cooling to room temperature, 4 mL of hexane was added for lipid extraction and vortexed for 1 min. After incubating at room temp for 10 min to permit phase separation, the entire hexane layer of each sample was transferred to a clean test tube. The hexane layer was evaporated using speed vacuum at −109 °C. Dried samples and standards were resuspended in 0.6 mL of hexane and transferred to autosampler vials for gas chromatography (GC) analysis.

#### 2.3.7. Gas Chromatography Methods

Cholesterol was determined using gas chromatography (Shimadzu GC-2014, Kyoto, Japan) with a flame ionized detector (FID) and an autosampler [49]. The separation was completed using an SPB-1 column (15 m × 0.32 mm i.d.; film thickness 1.0 mm) (Supelco Inc., Bellefonte, PA, USA) using helium as a carrier gas at a flow rate of 2 mL/min. The oven temperature was set at 285 °C, injection port temperature at 300 °C, and flame ionization detector temperature at 300 °C. The injection volume was 1 µL with a split ratio of 20:1. Matrix effects were addressed by the addition of an internal standard of 5-α-cholestane to all samples and standards. In addition, extracting the standards for each set of experiments using the same process as for the samples helped account for any errors in the preparation process. This allowed for the determination of the limits of detection (LOD) and quantitation (LOQ) for the experimental conditions of 8.31 µg/mL and 27.71 µg/mL, respectively.

#### 2.3.8. Calculations for Cholesterol Assimilation 

The integrated peak areas for cholesterol and the internal standard 5-α-cholestane were used to determine a 6-point calibration curve for cholesterol and used to extrapolate cholesterol recovered. The experimental calibration curve to determine LOD and LOQ were created by combining the calibration curves from five experiments to generate a linear calibration curve with R^2^ = 0.9832. Cholesterol assimilated and % cholesterol assimilated were calculated as follows, where Cholesterol*_i_* represents cholesterol content recovered at t = 0 and Cholesterol*_f_* represents cholesterol content recovered at given time point.

(1) $$\mathrm{Cholesterol}\;\mathrm{assimilated}={\;\mathrm{Cholesterol}}_{i}\;-\;{\mathrm{Cholesterol}}_{f}\;$$

(2) $$\%\;\mathrm{Cholesterol}\;\mathrm{assimilated}=\;\frac{\mathrm{Cholesterol}\;\mathrm{assimilated}}{{\mathrm{Cholesterol}}_{i}}\;\times \;100\%$$

### 2.4. Citrinin Production

#### 2.4.1. Citrinin Reagents

Citrinin, high-performance liquid chromatography (HPLC)-grade, was purchased from Sigma-Aldrich. A stock solution of citrinin at 100 ug/mL was prepared in HPLC-grade methanol (J.T. Baker) and used to construct a 7-point calibration curve as described in Section 2.4.3. Acetonitrile and water used in chromatography were HPLC grade (J.T. Baker), and trifluoroacetic acid (TFA) (Sigma-Aldrich, St. Louis, MO, USA) was analytical grade.

#### 2.4.2. Culture Preparation and Extraction for Citrinin Production

A 5 mm agar plug of *M. purpureus* was pre-cultured at 150 rpm and 30 °C for 4 days, and transferred to 10 mL of MEA + 0.3% or PBS + 0.3% oxgall and grown at 60 rpm and 37 °C, as described in culture preparations for cholesterol assimilation assays Section 2.3.2. At 24 h, 72 h, and 14 days, cultures were extracted for citrinin as described in Liu and Xu, with some modifications [50]. Briefly, 10 mL cultures were dounced and extracted with 10 mL of ethanol (1:1). Samples were then vortexed for 5 min and sonicated for 20 min. Samples were spun down at 4200× *g* for 10 min. Supernatant was collected, dried down, and resuspended in 1 mL HPLC-grade methanol. Extraction method was validated with recovery controls, where *M. purpureus* grown in MEA + 0.3% oxgall at 24 h and 72 h were spiked with citrinin at 10 μg/mL and extracted as described previously.

#### 2.4.3. High-Performance Liquid Chromatography Methods

Citrinin was determined using high-performance liquid chromatography, HPLC (Agilent 1100 liquid chromatograph) with a diode array detector (DAD) and an autosampler. The separation was completed using a Discovery C18 column (5 μm, 150 × 4.6 mm column) (Supelco Inc., Bellefonte, PA, USA) and an isocratic elution. The mobile phase consisted of acetonitrile:water containing 0.05% TFA and the volume ratio was 35:65 [50,51]. All samples, standards, and solvents were filtered through 0.22 μm membrane filters prior to HPLC analysis. The flow rate was 1 mL/min, and 20 μL sample was injected. The UV-DAD detection was monitored at 254 nm and 334 nm. The integrated peak areas at 334 nm for standard citrinin were used to determine a 7-point calibration curve and used to extrapolate citrinin recovered. The LOD and LOQ for the experimental conditions were 1.11 μg/mL and 3.70 μg/mL, respectively. Recovery of citrinin was determined by dividing citrinin concentration recovered by known citrinin concentration injected.

### 2.5. Statistical Analysis 

For cholesterol assimilation and growth curves, growing, resting, and dead *M. purpureus* cultures and controls were conducted in triplicate for each time point. For citrinin assays, growing and resting *M. purpureus* cultures were conducted in duplicate for each time point. All GC-FID and high-performance liquid chromatography-ultraviolet (HPLC-UV) samples were measured in duplicate. Two-way ANOVA was carried out to examine the effect of *M. purpureus* × incubation time interaction on growing, resting, or dead conditions. Tukey’s test was used to compare means. Significance was defined at *p* < 0.05 or *p* < 0.01. Standard deviation is calculated as either absolute error, or percent error through propagation of uncertainty from Equation (2) of Section 2.3.8. All statistical analyses were carried out using GraphPad Prism 8.0. 

## 3. Results

### 3.1. Cholesterol Assimilation 

The in vitro removal of cholesterol by the filamentous fungi *M. purpureus* CBS 109.07 (hereon referred to in the Results section as *M. purpureus*) was analyzed at 37 °C in media containing 0.3% (*w*/*v*) oxgall and 120 µg/mL cholesterol. Three growth phases were assessed: growing, where culture is active in MEA media; resting, where culture is dormant in PBS buffer; and dead, where culture has been heat-killed and incubated in MEA media. At indicated time points, an aliquot of spent media was collected from three independent replicates.

**Figure 1:**
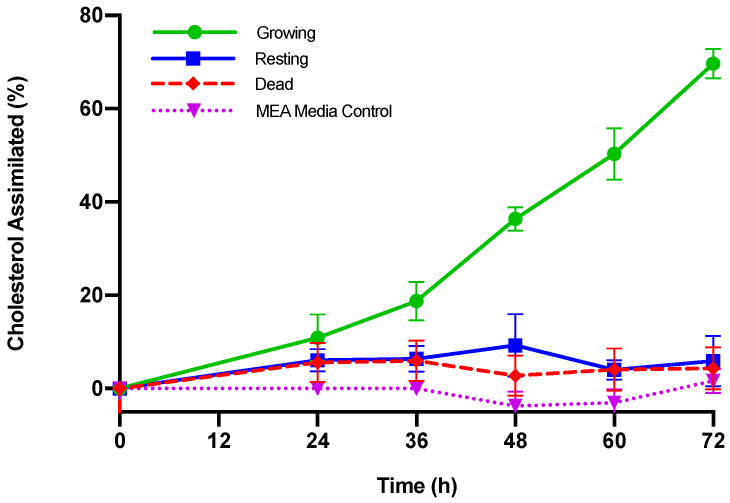
Cholesterol assimilated (%) by *M. purpureus* CBS 109.07 at different growth phases. Growing, resting, and dead *M. purpureus* cultures were incubated with 120 µg/mL cholesterol and 0.3% (*w*/*v*) oxgall bile salts at 37 °C. Malt extract media (MEA) control contained 120 µg/mL cholesterol and 0.3% (*w*/*v*) oxgall bile salts incubated without *M. purpureus*. Three independent trials were conducted for each condition at each time point, and cholesterol in samples were measured in duplicate via GC-FID. Standard deviation in cholesterol assimilated is percent error.

After 36 h, *M. purpureus* removed 18.69 µg/mL or 18.78% of the cholesterol in spent media, which is a significant decrease from the initial concentration (Table 1, Figure 1, *p* < 0.01). The rate of cholesterol removal was most dramatic from 36 to 60 h, and cholesterol removed increased from 18.78% to 50.27% (*p* < 0.05). At 72 h, 69.65% cholesterol was removed.

In contrast, *M. purpureus* cultures dormant in PBS for resting phase or heat-killed in dead phase removed a negligible amount of cholesterol after 72 h (Table 1) (*p* > 0.05). Non-growing *M. purpureus* conditions removed less than 10% of cholesterol, with a high percent error accounting for the propagation of the standard deviation. The media control containing MEA or PBS incubated without *M. purpureus* similarly showed no significant change in cholesterol content or cholesterol assimilation over 72 h (Table S1). We note that cholesterol content of resting and dead *M. purpureus* and media controls did not change significantly from the initial concentration of cholesterol within each trial (*p* < 0.01).

### 3.2. Growth of M. purpureus

The morphology of *M. purpureus* in submerged media is a compact, smooth, and spherical pellet consisting of intertwined hyphae [52,53,54]. Consistent with studies on other filamentous fungi, we found that dry weight measurement is the most reproducible method of quantifying *M. purpureus* growth [55,56,57,58,59,60,61].

**Figure 2:**
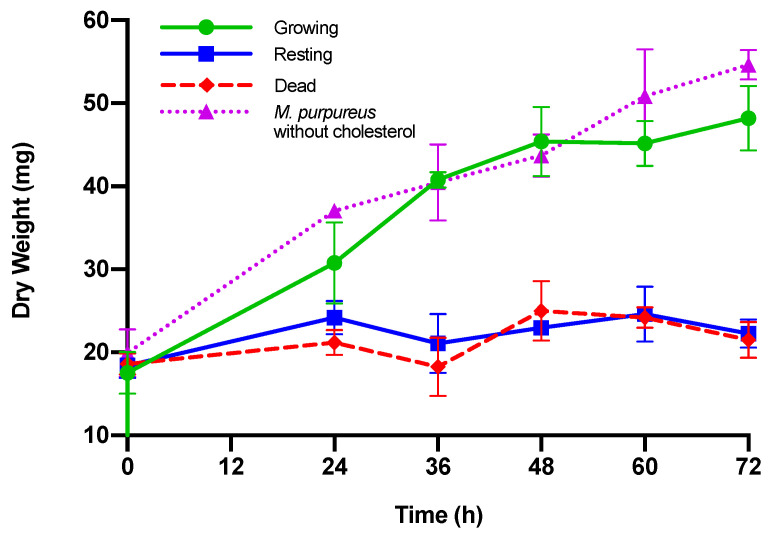
Dry weight of *M. purpureus* CBS 109.07. Growing, resting, or dead *M. purpureus* cultures were incubated in 120 µg/mL cholesterol and 0.3% (*w*/*v*) oxgall bile salts at 37 °C. *M. purpureus* incubated without cholesterol served as a control. Three independent trials were conducted for each growth phase at each time point. Standard deviation is represented by error bars.

After an aliquot of spent media was collected from three independent replicates, the remaining culture was harvested to measure dry weight (Table 2, Figure 2). Growth phases growing, resting, and dead were assessed and conditions included 0.3% oxgall and 120 µg/mL cholesterol. *M. purpureus* control was grown in MEA media without cholesterol. The dry weight of growing *M. purpureus* was significantly different from that of resting or dead *M. purpureus* (*p* < 0.05). The presence of cholesterol did not significantly enhance or inhibit the growth of *M. purpureus* [62] (*p* > 0.05). In both resting and dead conditions, *M. purpureus* had no significant growth (*p* > 0.05).

### 3.3. Reactivating Dormant *M. Purpureus*

*M. purpureus* incubated in PBS buffer, pH 7 with 0.3% oxgall and 120 µg/mL cholesterol does not assimilate cholesterol during a 96 h incubation (Table 3). To examine if resting conditions correspond to a dormant *M. purpureus*, cultures incubated in PBS were washed and transferred to MEA media with 0.3% oxgall and 120 µg/mL cholesterol. After four days of incubation in MEA, cholesterol assimilation was measured, but was not significantly different than MEA media control (*p* > 0.05). After seven days of incubation, cholesterol assimilation was initiated and cholesterol content is comparable to growing phase *M. purpureus* (Table S1). Dry weight of rescued *M. purpureus* was increased from resting *M. purpureus* (Table 2). The length of time incubated in PBS before the transfer to MEA did not have a significant effect on the ability to assimilate cholesterol (*p* < 0.05).

To determine if cholesterol is catabolized by *M. purpureus* as a carbon source, resting phase cultures in PBS were incubated with a nitrogen source, either ammonium sulfate or yeast nitrogen base without amino acids, at concentrations found in minimal media. We observed that resting *M. purpureus* in PBS supplemented with nitrogen sources had no significant cholesterol assimilation throughout a 24 and 72 h incubation (Table 4, *p* > 0.05). 

Fungi samples were then washed twice and transferred to MEA + 0.3% oxgall. The dry weight at day 4 in MEA of *M. purpureus* previously incubated in yeast nitrogen base was a significant increase from the initial weight before rescue (Table 5, *p* < 0.05), supporting the observation that dormant *M. purpureus* can be reactivated.

### 3.4. Citrinin Production in M. purpureus

The production of the mycotoxin citrinin was measured in *M. purpureus* grown under conditions used in cholesterol assimilation assays, where a 5 mm agar plug of *M. purpureus* was precultured in MEA at 150 rpm and 30 °C and then transferred to fresh media with oxgall and incubated at 60 rpm and 37 °C (Table 6). Samples collected at 24 h and 72 h did not have citrinin production above the limit of detection. The culture broth in these samples were also colorless. After 14 days, *M. purpureus* grown in MEA + 0.3% oxgall began to produce red pigment and culture broth turned reddish. *M. purpureus* in resting phase did not become pigmented. Though 14 days is outside the incubation period for this study’s cholesterol assimilation experiments, we extracted the red cultures, and measured 6.77 ± 1.02 µg citrinin per mL of culture broth. To validate extraction methods and show effectiveness, a set of *M. purpureus* cultures grown in MEA + 0.3% oxgall at 24 h and 72 h were spiked with citrinin at 10 μg/mL and extracted. Recovery of citrinin was 72.9% ± 3.8.

## 4. Discussion

As a natural source for monacolins, fermentation products of *Monascus purpureus* are widely used as alternative treatments for hypercholesterolemia. To the best of our knowledge, our data is the first to show that a strain of *M. purpureus* is capable of a cholesterol-lowering mechanism separate from its ability to produce monacolins and other secondary metabolites. We observed that active growing *M. purpureus* CBS 109.07 can assimilate cholesterol in vitro, and after 48 h incubation at 37 °C and high bile salt conditions, 36.38% of cholesterol content was removed. The removal of cholesterol by resting or dead *M. purpureus* CBS 109.07 was not statistically significant, and cholesterol was not catabolized as a carbon source. When resting cultures were washed and transferred to MEA media, *M. purpureus* CBS 109.07 became active and cholesterol assimilation and growth were observed. Citrinin production of *M. purpureus* CBS 109.07 incubated in growing or resting phase conditions at 24 h and 72 h was lower than the limit of detection, and we note that CBS 109.07 produced citrinin under our experimental conditions at day 14 when red pigment production was observed.

The ability of microorganisms to remove cholesterol in vitro from growth media is an indicator of probiotic potential, and the range of reduction percentage is wide and dependent on strain. We note that as with any therapeutic dosage, the concentration of microorganisms present will play a major role in cholesterol assimilation percentage. Miremadi et al. tested strains of *Lactobacilli* and *Bifidobacteria* and found 14 strains capable of removing cholesterol with a range of 34–65% assimilation after 24 h [41]. Eukaryotes capable of lowering cholesterol include strains of *S. boulardii*, *S. cerevisiae*, and *I. orientalis*, which after 48 h of incubation was observed to assimilate 90.6%, 96.8%, and 88.1% of cholesterol, respectively [37]. Strains of *P. kudriazevii*, *Galactomyces* sp., and *Y. lipolytica* were observed to assimilate 45.7%, 36.3%, and 30.9% of cholesterol, respectively, after 48 h of incubation in Chen et al. [45]. In the same study, the commercially available yeast probiotic *S. boulardii* lowered cholesterol by 36.5% at 48 h, and 41.5% cholesterol at 72 h. In this study, active growing *M. purpureus* CBS 109.07 was comparable to *S. boulardii* and was able to lower cholesterol from the media by 36.38% at 48 h, and 69.65% cholesterol at 72 h (Table 1). We note that at higher aeration and agitation, *M. purpureus* CBS 109.07 was able to assimilate a higher percentage of cholesterol (Table S2). When *M. purpureus* CBS 109.07 is resting or dead, cholesterol removal is not significant (Figure 1) and ranged from 2.75–9.29% removal after 72 h of incubation (Table 1). Other studies observed similar trends where resting and heat-killed cultures did not significantly reduce cholesterol [41,63,64,65], suggesting a mechanism where actively growing strains are more efficient at removing cholesterol.

To eliminate the possibility that cholesterol assimilation by *M. purpureus* was an artifact of starvation and the uptake of available carbon sources, we allowed cultures to incubate undisturbed until the entire culture was collected at the designated time point. This procedure differed from other cholesterol assimilation studies, where one-tenth of the culture volume was removed at each time point and, thus, could significantly impact nutrient availability [38,39,41,63,65,66,67]. We note that in our methods, the presence of cholesterol at 120 µg/mL did not enhance or inhibit the growth of *M. purpureus* CBS 109.07 as measured by dry weight (Figure 2).

We investigated the ability of *M. purpureus* CBS 109.07 to transition out of microbial dormancy after one to four days of incubation in PBS. Resting phase cultures incubated in PBS with 120 µg/mL cholesterol did not show significant cholesterol assimilation (Table 3). When washed and transferred to MEA media with 120 µg/mL cholesterol, previously resting phase *M. purpureus* CBS 109.07 cultures were able to restore cholesterol assimilation and growth at day 7 of rescue (Table 3) at levels comparable to growing cultures (Table S1, Table 2). There was no significant difference in reactivation of cholesterol assimilation between cultures that were incubated for one day or four days in PBS. We also observed that *M. purpureus* CBS 109.07 incubated in PBS with cholesterol and supplemented with nitrogen sources showed insignificant cholesterol assimilation between 24 to 72 h (Table 4), and were able to grow after transfer into MEA media for four days (Table 5). These results on resting phase reveal that *M. purpureus* CBS 109.07 incubated in PBS is indeed dormant and that cholesterol is not significantly taken up as a carbon source during dormancy. Additionally, the absence of cholesterol assimilation in PBS with nitrogen sources supports the assertion that *M. purpureus* CBS 109.07 does not metabolize cholesterol (Table 4). We posit that such an absence of cholesterol removal may reflect a mechanism where growing *M. purpureus* CBS 109.07 is more efficient at assimilating cholesterol.

Microorganisms can utilize the cholesterol-lowering mechanisms of active assimilation and passive adhesion to decrease host absorption of intestinal cholesterol [21,36,38]. Our results suggest that *M. purpureus* CBS 109.07 is capable of an in vitro active assimilation mechanism by growing cells. The dense pellet morphology of *M. purpureus* CBS 109.07 has made it difficult to measure the cholesterol content of the cell membrane, as similarly noted in biosorbent studies on other filamentous fungi such as *Aspergillus niger* and *Penicillium* sp. L1 strains [55,56,57]. Follow-up experiments will be conducted to lyse the *Monascus* membrane and examine membrane cholesterol content. Other cholesterol-lowering mechanisms by probiotic microorganisms include modulation of lipid metabolism and deconjugation of bile salts [36]. *M. purpureus* is capable of directly modulating lipid metabolism, as it synthesizes monacolins that directly inhibit HMG-CoA reductase, the committed step of cholesterol biosynthesis in the liver [7,8]. In future studies, we will assay *M. purpureus* CBS 109.07 for bile salt hydrolase (BSH) activity, the enzyme responsible for the deconjugation of bile salts found in many probiotic strains [68,69].

Like many strains within *Monascus*, *Aspergillus*, and *Penicillium* genera, *M. purpureus* CBS 109.07 can biosynthesize citrinin, with levels highly dependent on the growth conditions and amount of microorganisms used. In this study, the citrinin production in *M. purpureus* CBS 109.07 under growing and resting phase conditions replicated from our cholesterol assimilation experiments was below our limit of detection of 1.11 µg/mL (Table 6). Our results at 24 h and 72 h are consistent with published studies on other *M. purpureus* strains which measured citrinin production in different growth conditions over time. These studies observed delays in citrinin production, with detection of citrinin beginning as early as day 5 or late as day 10 [70,71,72]. Notably, the commencement of citrinin production corresponded to the commencement of red pigment production, and increases in agitation and aeration increased citrinin production [72,73]. *M. purpureus* CBS 109.07 studies in particular did not measure citrinin at early time points. However using thin-layer chromatography (TLC), they reported citrinin level to be 5 µg/mL after 14 day incubation in glucose media and unspecified agitation [30], and 65 µg/mL after 7 day incubation in ethanol media and 220 rpm agitation [74,75]. We used HPLC-UV to quantify citrinin production after 14 day incubation in MEA + 0.3% oxgall and 60 rpm. At day 14 under our conditions, the culture broth began to turn reddish, and we measured a citrinin concentration of 6.77 µg/mL [30,74,75,76]. The differences in citrinin production between CBS 109.07 studies highlight how critical growth conditions are to the control of citrinin levels in *Monascus* strains [77,78]. In future studies, we can target the citrinin issue as many studies have successfully eliminated or reduced the levels of citrinin by disrupting the citrinin biosynthetic genes *pksCT* or *ctnA* in *M. purpureus* [70,79,80,81].

To be beneficial for human health, probiotic microorganisms must be capable of surviving transit through the human gastrointestinal tract. Absorption of dietary cholesterol into the bloodstream occurs predominantly in the duodenum of the small intestine, where the pH varies from pH 6 to 7 and bile salts excreted from the bile duct assist in solubilizing cholesterol [82,83]. *M. purpureus* CBS 109.07 was cultured in media at physiological temperature and pH, and with a high bile salt concentration and low aeration and agitation. However we recognize the limitations of an in vitro study in reproducing gastrointestinal conditions. Additionally, the clinical safety of *M. purpureus* CBS 109.07 needs to be established—a potentially complicated issue if the restrictive regulations on *Monascus* pigments and red yeast rice supplements by the FDA and European Food Safety Authority are any indication [4,5,31,84]. In the current study, we are only beginning to raise the possibility of an application for *M. purpureus* CBS 109.07 in probiotics; we recognize that additional safety and gastrointestinal survival experiments are required, and that such advancement in understanding *Monascus* biochemistry may improve the restrictions on their usage in the US and EU [85]. We also recognize that other candidate strains of *M. purpureus* or other *Monascus* species may be found [86], and that CBS 109.07 may not be unique or exemplary. However, we note that the human consumption of *M. purpureus* CBS 109.07 has precedents, as several food-grade studies have considered CBS 109.07 an edible filamentous fungus and used it as the representative *Monascus* strain in human and animal food applications of mycoprotein [6,47,48].

## 5. Conclusions

Our findings demonstrate that *M. purpureus* CBS 109.07, which can biosynthesize statin-like monacolins, can also reduce cholesterol content in vitro via a mechanism of cholesterol assimilation at 37 °C with a high concentration of bile salts. The most effective removal of cholesterol occurred in growing *M. purpureus* CBS 109.07 cultures, while non-growing *M. purpureus* CBS 109.07 minimally adhered to cholesterol and did not metabolize cholesterol. Dormant cultures, once transferred from buffer to nutrient rich media, were able to be resume cholesterol assimilation at levels observed in active cultures. Citrinin production under our experimental conditions was not detected. Our results show that it is valuable to continue examining the cholesterol-lowering potential of active *M. purpureus* CBS 109.07 cultures, as further research may provide a possible insight in the treatment of hypercholesterolemia and will draw attention to the significance of filamentous fungi in human health and nutrition. 

## Figures and Tables

**Table 1:** Cholesterol assimilated in *M. purpureus* CBS 109.07 at different growth phases. All cultures were incubated at 37 °C with 120 µg/mL cholesterol and 0.3% (*w*/*v*) oxgall bile salts. Cholesterol assimilated was calculated from initial cholesterol, and determined from three independent trials conducted for each growth phase at each time point, and measured in duplicate via gas chromatography-flame ionized detection (GC-FID). Standard deviation calculated is absolute error (µg/mL) and percent error (%).

	Cholesterol Assimilated					
Time (h)	Growing *M. purpureus*		Resting *M. purpureus*		Dead *M. purpureus*	
	(µg/mL)	(%)	(µg/mL)	(%)	(µg/mL)	(%)
0	--	--	--	--	--	--
24	10.89 ± 5.62 ^+^	10.91 ± 7.47	6.78 ± 2.79	6.07 ± 6.97	5.68 ± 4.40	5.59 ± 4.95
36	18.69 ± 4.53 ^a,+^	18.78 ± 7.03 ^a,+^	7.20 ± 3.33 ^b^	6.40 ± 6.02 ^b^	6.04 ± 4.54 ^b^	5.94 ± 5.02 ^b^
48	36.18 ± 3.84 ^a,+^	36.38 ± 5.38 ^a,+^	10.53 ± 7.94 ^b,+^	9.29 ± 7.25 ^b^	2.88 ± 4.45 ^c^	2.75 ± 4.69 ^b^
60	49.86 ± 5.30 ^a,+^	50.27 ± 13.5 ^a,+^	4.54 ± 2.48 ^b^	4.03 ± 6.03 ^b^	4.21 ± 4.63 ^b^	4.08 ± 4.71 ^b^
72	69.13 ± 3.95 ^a,+^	69.65 ± 12.5 ^a,+^	6.70 ± 6.33 ^b^	5.91 ± 7.23 ^b^	4.44 ± 4.67 ^b^	4.33 ± 5.16 ^b^

^a, b, c^ Means within a row are significantly different (*p* < 0.01). ^+^ Means significantly different from the initial value at t = 0 (*p* < 0.01).

**Table 2:** Dry weight of *M. purpureus* CBS 109.07. Growing, resting, or dead *M. purpureus* cultures were incubated in 120 µg/mL cholesterol and 0.3% (*w*/*v*) oxgall bile salts at 37 °C. *M. purpureus* incubated without cholesterol served as a control. Three independent trials, corresponding to samples used for cholesterol assimilation, were conducted for each growth phase at each time point.

	Dry Weight (mg)			
Time (h)	Growing *M. purpureus*	Resting *M. purpureus*	Dead *M. purpureus*	*M. purpureus* without Cholesterol
0	17.54 ± 2.5	18.40 ± 1.5	18.60 ± 1.3	19.91 ± 2.9
24	30.77 ± 4.9 ^a,+^	24.17 ± 2.0 ^a^	21.17 ± 1.5 ^b^	37.05 ± 0.5 ^c,+^
36	40.77 ± 0.9 ^a,+^	21.07 ± 3.5^b^	16.93 ± 5.6 ^b^	40.45 ± 4.6 ^a,+^
48	45.40 ± 4.2 ^a,+^	22.93 ± 0.5 ^b^	25.00 ± 3.6 ^b,+^	43.72 ± 2.5 ^a,+^
60	45.17 ± 2.7 ^a,+^	24.60 ± 3.3 ^b^	24.17 ± 1.3 ^b^	50.87 ± 5.6 ^a,+^
72	48.20 ± 3.9 ^a,+^	22.27 ± 1.7 ^b^	21.50 ± 2.2 ^b^	54.63 ± 1.8 ^a,+^

^a, b, c^ Means within a row are significantly different (*p* < 0.05). ^+^ Means significantly different from the initial value at t = 0 (*p* < 0.05).

**Table 3:** Cholesterol content (µg/mL) and dry weight (mg) of *M. purpureus* CBS 109.07 incubated in phosphate-buffered saline (PBS) and rescued in MEA. All cultures were incubated at 37 °C with 120 µg/mL cholesterol and 0.3% (*w*/*v*) oxgall bile salts. Resting *M. purpureus* was washed in PBS + 0.3% oxgall and then transferred to MEA with 0.3% oxgall and 120 µg/mL cholesterol, and sample collected after 4 and 7 days. Data were determined from three independent trials conducted at each time point. Cholesterol content was measured in duplicate via GC-FID. Standard deviation in cholesterol content is absolute error.

	PBS ^1^		Day 4 Incubation in MEA ^1^	Day 7 Incubation in MEA ^1^	Day 7 Incubation in MEA ^1^
Time (h)	Cholesterol Content (µg/mL)	Time ^2^ (h)	Cholesterol Content (µg/mL)	Cholesterol Content (µg/mL)	Dry Weight (mg)
24	114.46 ± 3.73 ^a^	24	97.73 ± 2.66 ^b^	38.83 ± 9.95 ^c,+^	32.87 ± 2.9 ^++^
48	118.30 ± 3.12 ^a^	48	97.12 ± 4.40 ^b^	40.88 ± 9.51 ^c,+^	31.70 ± 2.7 ^++^
72	117.36 ± 6.84 ^a^	72	98.36 ± 2.10 ^b^	37.24 ± 11.47 ^c,+^	31.60 ± 4.9 ^++^
96	114.81 ± 3.86 ^a^	96	98.63 ± 8.19 ^b^	52.23 ± 5.30 ^c^	25.33 ± 2.0

^1^ Media contained 0.3% oxgall and 120 µg/mL cholesterol. ^2^ Time corresponds to duration incubated in PBS prior to rescue in MEA media. ^a, b, c^ Cholesterol content means within a row are significantly different (*p* < 0.05). ^+^ Means significantly different from the MEA media control Table S1 (*p* < 0.05). ^++^ Means significantly different from the resting *M. purpureus* dry weight Table 2 (*p* < 0.05).

**Table 4:** Cholesterol content (µg/mL) of *M. purpureus* CBS 109.07 incubated in PBS with nitrogen sources. All cultures were incubated at 37 °C with 120 µg/mL cholesterol and 0.3% (*w*/*v*) oxgall bile salts. Cholesterol content was determined from two independent trials conducted for each growth phase at each time point, and measured in duplicate via GC-FID. All means were not significantly different from the initial value at t = 0 (*p* < 0.05). Standard deviation in cholesterol content is absolute error.

	Cholesterol Content of *M. purpureus* Incubated in PBS with 0.3% Oxgall (µg/mL)	
Time (h)	With Yeast Nitrogen Base without Amino Acids	With Ammonium Sulfate
0	105.82 ± 2.20	105.46 ± 4.59
24	97.91 ± 2.03	89.64 ± 2.59
72	97.57 ± 1.60	93.45 ± 8.03

**Table 5:** Dry weight (mg) of *M. purpureus* CBS 109.07 after incubation in PBS with 120 µg/mL cholesterol and 0.3% (*w*/*v*) oxgall bile salts and rescued in MEA with 0.3% (*w*/*v*) oxgall bile salts. *M. purpureus* is washed in PBS + 0.3% oxgall and then transferred to MEA with 0.3% oxgall, and sample collected after 4 days. Two independent trials, corresponding to samples used in PBS cholesterol assimilation, were conducted for each growth phase at each time point. Standard deviation in cholesterol content is absolute error.

	Dry Weight of *M. purpureus* (mg)			
	PBS + 0.3% Oxgall		Day 4 Incubation in MEA + 0.3% Oxgall	
Time ^1^ (h)	With Yeast Nitrogen Base	With Ammonium Sulfate	With Yeast Nitrogen Base	With Ammonium Sulfate
0	19.4 ± 3.1 *	18.8 ± 3.1 *		
24			37.5 ± 2.8 ^+^	34.7 ± 3.3 ^+^
72	25.8 ± 5.3	23.3 ± 3.2	39.3 ± 0.4 ^+^	30.8 ± 1.8 ^+^

^1^ Time corresponds to duration incubated in PBS prior to rescue in MEA media. * Initial weight at t = 0. ^+^ Means significantly different from the initial weight at t = 0 (*p* < 0.05).

**Table 6:** Citrinin production under experimental conditions. Citrinin concentration (µg/mL) of *M. purpureus* CBS 109.07 under experimental conditions of growing and resting phases was measured at 24 and 72 h via HPLC-UV. Two independent trials were conducted for each time point. Standard deviation in cholesterol content is absolute error.

Citrinin Production of *M. purpureus* (µg Citrinin/mL of Culture Broth)			
Media Conditions	After 24 h ^1^	After 72 h ^1^	After 14 Days
MEA + 0.3% oxgall	ND	ND	6.77 ± 1.02
PBS + 0.3% oxgall	ND	ND	

^1^ ND is not detected with a peak below the limit of detection (LOD) of 1.11 µg/mL.

## Supplementary Materials

The following are available online at https://www.mdpi.com/2309-608X/6/4/352/s1, Table S1: Cholesterol content of *M. purpureus* CBS 109.07, Table S2. Cholesterol content, cholesterol assimilated, and dry weight of growing *M. purpureus* CBS 109.07.
