# Supplementary material for: Assimilation of Cholesterol by Monascus purpureus
Source: J Fungi (Basel). 2020 Dec 9;6(4):352. doi: 10.3390/jof6040352 (PMC7770578; doi:10.3390/jof6040352)
Supplement: Supplementary file 1 [file jof-06-00352-s001.zip › Supplemental Materials Revision - jof1003481/Nguyen et al. - Supplemental Materials-Revisions.docx]

Supplemental Materials

Assimilation of cholesterol by *Monascus purpureus*

Theresa P.T. Nguyen ^*^, Margaret A. Garrahan ^†^, Sabrina A. Nance ^†^, Catherine E. Seeger ^†^ and Christian Wong ^†^

Loyola University Maryland, Department of Chemistry & Biochemistry, Baltimore, MD, USA.

***** Correspondence: [tptnguyen@loyola.edu](mailto:tptnguyen@loyola.edu); Tel: 1-410-617-2862

^†^ These authors contributed equally to this work.

1. Materials and Methods

1.1. Strains and Media Conditions

*M. purpureus* Went teleomorphic type strain CBS 109.07 (ATCC 16365) was obtained from the ATCC strain bank and chosen for its ability to grow at 30 °C. *M. purpureus* was grown in a malt extract media (MEA) containing: 2% soluble Bactomalt extract (BD Bioscience), 2% glucose, 1% peptone, and pH adjusted to pH 7. Plate media contained 2% agar. Phosphate-buffered saline solution (PBS) contained 8.0 g NaCl, 0.2 g KCl, 1.44 g Na_2_HPO_4_, 0.24 g KH_2_PO_4_ for every 1.0 L solution and was pH adjusted to pH 7. Bile salt supplemented media contained 0.3% (w/v) oxgall (BD Difco). Strains were maintained as described in Nguyen et al. in section 2.2.

1.2. Cholesterol Assimilation for Table S1

Growing, resting, and *M. purpureus* control conditions contained *M. purpureus* CBS 109.07 pellets that were homogenized using a sterilized glass douncer in a sterile 50 mL conical tube and divided into replicates. Dead culture conditions contained *M. purpureus* CBS 109.07 pellets that were autoclaved at 121 °C for 20 min under 15 psi pressure and transferred to fresh media. Growing and dead cultures contained 10 mL MEA media in sterile 50 mL borosilicate tubes; resting cultures contained 10 mL of PBS in sterile 50 mL borosilicate tubes. Cholesterol assimilation and dry weight experiments were supplemented with 0.3% oxgall and incubated at 37 °C and 60 rpm. With the exception of the *M. purpureus* control, all conditions were incubated with 120 µg/mL cholesterol. Media control without *M. purpureus* contained 120 µg/mL cholesterol and 0.3% (w/v) oxgall in 10 mL MEA or 10 mL PBS.

Cholesterol assimilation and dry weight experiments for each growth condition were prepared in triplicate, where three independent sets of cultures were homogenized and divided into six sterile 50 mL borosilicate tubes to account for six timepoints (0, 24, 36, 48, 60, and 72 h). At designated time point, a 1.0 mL aliquot of culture supernatant was collected, centrifuged at 2000 x *g* for 15 min, and stored in a 50 mL borosilicate glass tube with PTFE lined cap at -20 °C. Cholesterol quantification was performed as described in Nguyen et al. sections 2.3.6 to 2.3.8.

1.3. Cholesterol Assimilation and Dry Weight for Table S2.

Cholesterol assimilation experiments were collected from three independent samples. Unlike previous cholesterol assimilation experiments described in Nguyen et al., this method collected one-tenth of the volume at each timepoint. Growing conditions contained *M. purpureus* CBS 109.07 pellets in 10 mL MEA media in 50 mL borosilicate tubes. All cholesterol assimilation was supplemented with 0.3% oxgall and 225 µg/mL and incubated at 37 °C and 150 rpm. A 1.0 mL aliquot of culture supernatant was collected at the starting point (t = 0 h) and after 24, 36, and 48 h. Samples were stored in a 50 mL borosilicate glass tube with PTFE lined cap at -20 °C. Cholesterol quantification was performed as described in Nguyen et al. sections 2.3.6. to 2.3.8.

Dry weight experiments were independent from cholesterol assimilation samples and prepared in triplicate for each time point in sterile 50 mL borosilicate tubes. At designated time points, the complete contents of the borosilicate tube was harvested and filtered via vacuum flask and a Whatman filter #4. The contents were allowed to air dry for 5 days and weighed on an analytical balance.

**Supplemental Table 1.** Cholesterol content (µg/mL) and cholesterol assimilated (µg/mL) in *M. purpureus* CBS 109.07 at different growth phases. All cultures incubated at 37 ºC with 120 µg/mL cholesterol and 0.3% (w/v) oxgall bile salts. Cholesterol content was determined from three independent trials conducted for each growth phase at each time point, and measured in duplicate via GC-FID. Standard deviation in cholesterol content is absolute error.

|  |  | Cholesterol Content (µg/mL) | | | | | | |
| --- | --- | --- | --- | --- | --- | --- | --- | --- |
|  |  |  |  |  |  | Media Control | | |
| Time (h) |  | Growing  *M. purpureus* | Resting  *M. purpureus* | Dead  *M. purpureus* |  | MEA + 0.3% oxgall | PBS + 0.3% oxgall | |
| 0 |  | 99.28 ± 4.33 | 111.44 ± 5.19 | 99.22 ± 4.44 |  | 103.52 ± 4.27 | 124.59 ± 1.36 | |
| 24 |  | 88.40 ± 5.36^+^ | 104.66 ± 5.42 | 99.54 ± 1.98 |  | 119.36 ± 2.31^+^ | | 126.75 ± 1.98 |
| 36 |  | 80.59 ± 4.44^+^ | 104.24 ± 3.97^+^ | 93.19 ± 2.12 |  | 114.48 ±1.30^+^ | 122.48 ± 3.69 | |
| 48 |  | 63.01 ± 1.98^+^ | 100.91 ± 5.61^+^ | 96.34 ± 1.33 |  | 109.07 ± 5.91 | 130.80 ± 1.99 | |
| 60 |  | 49.42 ± 6.31^+^ | 106.90 ± 4.09 | 95.02 ± 1.37 |  | 106.57 ± 2.61 | 124.50 ± 5.08 | |
| 72 |  | 30.15 ± 3.53^+^ | 104.73 ± 5.79 | 94.78 ± 2.44 |  | 101.72 ± 3.12 | 127.98 ± 1.91 | |

^+^ Means significantly different from the initial value at t = 0 (p < 0.01)


**Supplemental Table 2.** Cholesterol content (µg/mL) and cholesterol assimilated (%) in *M. purpureus* CBS 109.07. All cultures were incubated at 37 ºC with 225 µg/mL cholesterol and 0.3% (w/v) oxgall bile salts and 150 rpm. Cholesterol content was determined from three independent trials and measured in duplicate via GC-FID. Standard deviation in cholesterol content and dry weight is absolute error, and standard deviation in cholesterol assimilated is percent error.

|  |  | Growing *M. purpureus* | | | | |
| --- | --- | --- | --- | --- | --- | --- |
| Time (h) |  | Cholesterol Content  (µg/mL) |  | Cholesterol Assimilated  (%) |  | Dry Weight  (mg) |
| 0 |  | 170.63 ± 7.83 |  | -- |  | 15.0 ± 2.6 |
| 24 |  | 132.27 ± 7.87 |  | 22.28 ± 7.51% |  | 31.9 ± 7.5^+^ |
| 36 |  | 119.93 ± 7.04^+^ |  | 29.71 ± 7.45% |  | 38.6 ± 3.5^+^ |
| 48 |  | 76.33 ± 2.31^+^ |  | 55.27 ± 5.50% |  | 47.2 ± 4.8^+^ |

^+^ Means significantly different from the initial value at t = 0 (p <0.05)
